# Lymph node status in screen-detected cancers

**DOI:** 10.1038/sj.bjc.6602290

**Published:** 2005-01-14

**Authors:** S W Duffy

**Affiliations:** 1 Cancer Research UK Centre for Epidemiology, Mathematics and Statistics, Wolfson Institute of Preventive Medicine, Charterhouse Square, London EC1 M 6BQ, UK

In this issue, Bucchi *et al* (2005) report an interaction between detection mode (screening or clinical), tumour size and lymph node status in breast cancer that is of considerable interest in understanding the screening mechanism. Its potential importance can be seen when expressed as follows: in 1842 invasive tumours of size 2–17 cm, 14% of screen-detected cases were node positive, whereas in clinically detected cases, more than double that proportion, 29%, were node positive; on the other hand, in 1487 tumours of size 18 mm or more, the proportions of node positive tumours were almost equal, at 45 and 50% for screen- and clinically detected cancers, respectively. Thus, the advantage in terms of node status conferred by screen detection is only observed in the small tumours.

Length bias may partly explain this phenomenon, in that the smaller screen-detected tumours are more likely to be slow-growing cases than the larger. However, this is unlikely to explain an interaction of the magnitude observed here. The authors conclude that this is evidence that the biological aggressiveness of a breast tumour increases as the tumour develops, notably during its subclinical phases. This is consistent with evidence that histological grade may deteriorate with tumour development, at least in some tumours (Tabar *et al*, 1996).

The results of Bucchi *et al* can also be expressed in terms of the effect of size on node status within screen- and clinically detected tumours separately. In clinically detected tumours, the odds ratio of node positive disease for tumours size 28 mm or more compared to those of size 2–7 mm is 9.6. In screen-detected tumours, the corresponding odds ratio is approximately double this figure at 18.9. Thus, the dependence of node status on tumour size is much stronger in screen-detected tumours.

The results will surely prompt others to look again at their own tumour data. Some time ago, Tabar *et al* (1987) found no such interaction in 964 cancers in one county of the Swedish Two-county Trial of breast cancer screening. It will be of great interest to see the results of a more statistically powerful analysis of node status by tumour size and detection mode in the updated tumour population of 2299 invasive cancers in these counties (Tabar *et al*, 2000).

The practical implication for those involved in breast cancer screening is to further emphasise the importance of detecting the tumour while it is small. Although node status is clearly an important determinant of final outcome (Balslev *et al*, 1994) and of the success of screening in altering this outcome (Smith *et al*, 2004), the results of Bucchi *et al* show that the opportunity for screening to lead to detection and treatment before spread to the lymph nodes is extremely limited once the tumour has grown to 20 mm or more in diameter.

Finally, there is another very interesting aspect to this paper, namely the categorisation of tumour size. Noting the tendency to render this as discrete multiples of 5 mm, the authors chose the categories as 2–7, 8–12 mm, and so on, so that each class represents a cluster around 5, 10 and higher multiples of five. This is a brave but eminently sensible departure from the convention of choosing cutoffs at multiples of 5 or 10 mm, which brings with it the decision whether to include the cutoff point in the lower group, as in the TNM convention or in the upper group, as practised by some researchers. An alternative to categorisation is to use individual values of tumour size as continuous measures, with spline or fractional polynomial regression analyses (Royston *et al*, 2000), but this depends on how confident we are in a heavily parametric model of the dependence of node status on size and in the continuous nature of the tumour size data. Figure 1 of Bucchi *et al* shows that for the most part, we do not have continuous tumour size data, but discrete scoring of the underlying continuous phenomenon of size. In such a case, it makes sense to categorise the data around the discrete values recorded.
